# SynBio2Easy—a biologist-friendly tool for batch operations on SBOL designs with Excel inputs

**DOI:** 10.1093/synbio/ysac002

**Published:** 2022-01-26

**Authors:** Tomasz Zieliński, Johnny Hay, Andrew Romanowski, Anja Nenninger, Alistair McCormick, Andrew J Millar

**Affiliations:** SynthSys & Institute of Molecular Plant Sciences, School of Biological Sciences, University of Edinburgh, Edinburgh, UK; EPCC, University of Edinburgh, Edinburgh, UK; SynthSys & Institute of Molecular Plant Sciences, School of Biological Sciences, University of Edinburgh, Edinburgh, UK; SynthSys & Institute of Molecular Plant Sciences, School of Biological Sciences, University of Edinburgh, Edinburgh, UK; SynthSys & Institute of Molecular Plant Sciences, School of Biological Sciences, University of Edinburgh, Edinburgh, UK; SynthSys & Institute of Molecular Plant Sciences, School of Biological Sciences, University of Edinburgh, Edinburgh, UK

**Keywords:** synthetic biology, SBOL, SynBioHub, research data management, software tools, spreadsheet

## Abstract

Practical delivery of Findable, Accessible, Reusable and Interoperable principles for research data management requires expertise, time resource, (meta)data standards and formats, software tools and public repositories. The Synthetic Biology Open Language (SBOL2) metadata standard enables FAIR sharing of the designs of synthetic biology constructs, notably in the repository of the SynBioHub platform. Large libraries of such constructs are increasingly easy to produce in practice, for example, in DNA foundries. However, manual curation of the equivalent libraries of designs remains cumbersome for a typical lab researcher, creating a barrier to data sharing. Here, we present a simple tool SynBio2Easy, which streamlines and automates operations on multiple Synthetic Biology Open Language (SBOL) designs using *Microsoft Excel®* tables as metadata inputs. The tool provides several utilities for manipulation of SBOL documents and interaction with SynBioHub: for example, generation of a library of plasmids based on an original design template, bulk deposition into SynBioHub, or annotation of existing SBOL component definitions with notes and authorship information. The tool was used to generate and deposit a collection of 3661 cyanobacterium *Synechocystis* plasmids into the public SynBioHub repository. In the process of developing the software and uploading these data, we evaluated some aspects of the SynBioHub platform and SBOL ecosystem, and we discuss proposals for improvement that could benefit the user community. With software such as SynBio2Easy, we aim to deliver a user-driven tooling to make FAIR a reality at all stages of the project lifecycle in synthetic biology research.

Graphical Abstract

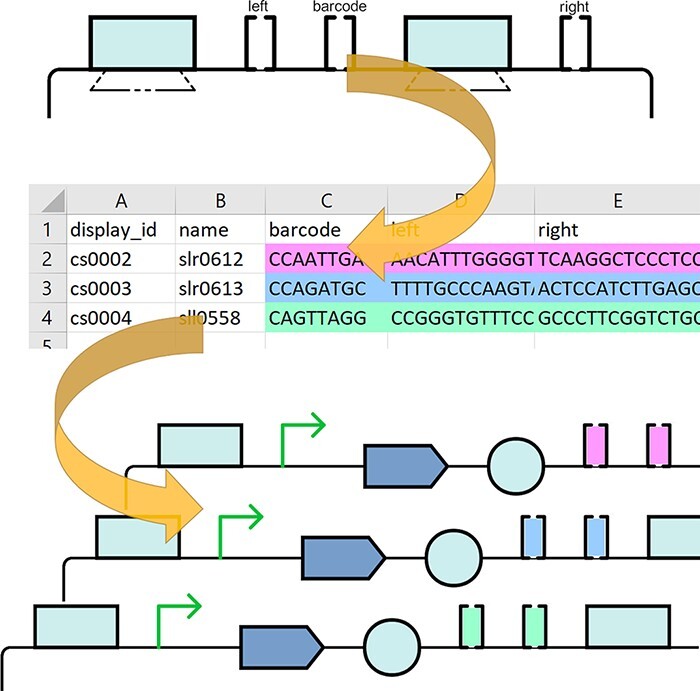

## 1. Introduction

As the open science landscape evolves, funding bodies and researchers themselves are developing ever-increasing expectations for opening access to the outcomes of their projects. The Synthetic Biology research community has a strong record and ethos of data sharing. Concomitantly, ever more rigorous standards have formed relating to open data; critically, how they are stored, documented, accessed and enriched with descriptive metadata. The FAIR data management practices are exemplary: promoting Findability, Accessibility, Interoperability and Reusability in scientific research data (1, 2). However, it is not always clear for researchers—whose focus is rightly on experimentation and generating meaningful results—how these recommendations can be attained. Therefore, it is vital that research communities invest in not only adopting these practices, but also crucially in facilitating the delivery of these standards and practices by researchers while presenting minimal friction with their primary work.

One of the most fundamental decisions researchers need to make in order to deliver effective, sustainable outputs with maximum impact is where they will publish and share their data. Candidate data repositories should encourage FAIR practices that will satisfy funders and serve the open science imperatives. They should also provide software tools, support and documentation that will empower researchers to perform their data management tasks with minimum efforts and preferably add value to the data they share. For synthetic biologists who design biological constructs, resources are available such as SynBioHub (3), or the Joint BioEnergy Institute’s public Inventory of Composable Elements (ICE), an open source registry software and platform for managing information about biological parts (4). We have previously evaluated both platforms and concluded that while they can be considered complementary solutions, SynBioHub shows more potential with its intrinsic handling of knowledge graphs (5). The SynBioHub repository offers a set of features that enable FAIR practices, as well as provides a flexible programmatic API with mature software tools and active community support.

The FAIR data principles are summarized as ‘Facets’: data should be Findable, Accessible, Reusable and Interoperable (2), and SynBioHub supports these in a number of ways. For findability, (meta)data are registered and indexed as Resource Description Framework (RDF) (6) documents in a searchable knowledge graph, which can be queried using the ‘SPARQL Protocol and RDF Query Language’ (SPARQL) (7). This provides a powerful querying interface and allows access to all the metadata stored about entities (5). Besides using standard protocols such as SPARQL, SynBioHub makes data accessible because it provides a user interface (UI) that is user-friendly, where (meta)data can be easily retrieved in multiple formats, and it also exposes an Application Programming Interface (API) for easy programmatic access. Design (meta)data stored in SynBioHub are represented using SBOL 2.0 (8), an open, standardized data model for interoperability. The rich metadata model created through SBOL is flexible and expressive, having a plurality of relevant attributes, while data deposited in SynBioHub can be downloaded in the native SBOL format or first converted into an alternative format such as GenBank (9) or FASTA (10), thus allowing the designs to be exported easily and reused by future researchers.

CyanoSource is a joint project between the McCormick lab at the University of Edinburgh, the Lea-Smith lab at the University of East Anglia, Edinburgh Genome foundry and Earlham Biofoundry. Among the outcomes of this research, a collection of recombinant plasmids targeting up to 3661 genes of the cyanobacterium *Synechocystis* sp. PCC 6803 (*Synechocystis* hereafter) are being assembled (based on genome assembly ASM972v1 from Kazusa (11)). This project aims to significantly advance the development of strains for biotechnology applications, and adherence to FAIR is an effective way to achieve it. The plasmids have been designed and now they are starting to be produced in batches by Golden Gate Assembly, checked and finally transformed into *Synechocystis* to induce a single gene deletion by homologous recombination. During the project, the plasmids will go through the following stages:

Stage 1—the initial plasmid designs, waiting for production.

Stage 2—the assembled plasmids. In some cases, they have a different structure than the initial designs from stage 1. If nothing else, their descriptions are updated to reflect the status change to ‘produced’.

Stage 3—plasmids are verified by sequencing (also to check the barcodes are correct) and the real sequences are attached.

Stage 4—plasmids are transformed into *Synechocystis* and checked by PCR to see if (i) the target gene has been knocked out and (ii) if the strain has fully segregated (i.e. all copies of the genome in the strain contain the gene deletion). This information is also recorded with the description.

All of these designs are based on the same template pUC19 plasmid backbone and selection markers (codA and KanR), with variations in the combinations of sequences in homology flanks (i.e. the sequences flanking the gene sequence to be removed) and barcode markers (12). In our earlier evaluation of the SynBioHub platform (5), it was clear that the repository is ideal for publication of this type of data. To leverage the best available features of SynBioHub, however, it would be necessary to utilize the SBOL standard to describe the designs. Evidently, expecting researchers to manually create SBOL designs on this scale would be totally impracticable. It was therefore necessary to develop a software tool that would create an original template plasmid document and then generate a derived design for each variation of flank and barcode sequences. Once the library of plasmids was created, they would need to be deposited into SynBioHub for publication, which again would be a perfect task to automate. Finally, as the project transitions through the stages outlined above, there would need to be a convenient mechanism for performing metadata updates on multiple records.

There is a rich ecosystem of SBOL-related tools that help in creating designs such as SBOLCanvas (13), SBOLDesigner (14), ShortBOL (15) or Cello (16). However, some of these were not suitable for the requirements of this project because they are intended for biologists to craft a single design at a time in a CAD-like environment (SBOLDesigner) or GUI (SBOLCanvas) and could not easily create multiple designs. Other tools, such as ShortBOL and Cello, require understanding of domain-specific languages or rules-based programming, which was unsuitable for our users who do not have the resources to dedicate to learning these frameworks.

It was clear from early on in our project that MS Excel (17) would be the optimal data input format. Biologists routinely use Excel for recording and storing their designs, so the necessary inputs are typically already available in this format. Using Excel tables has the dual benefit that users feel comfortable with a familiar tool, while providing an effective but compact format to represent information about hundreds of synthetic designs. We therefore decided to provide the plasmid sequences and metadata attributes inside Excel spreadsheets, while the software itself is designed to accept various operational parameters such as the path to the Excel file, target SynBioHub collection URL and user credentials. While development of our software progressed, it emerged that another similar tool was in development: Excel2SBOL, an open-source Python library for converting MS Excel templates into SBOL documents (18). Although it was not available at the inception of our project, the release of Excel2SBOL reaffirms the utility of tools that can generate SBOL documents at scale.

## 2. Materials and methods

The SynBio2Easy application was written in the Java v11 (19) programming language. There are two main components that comprise the software tool: the SBOL2Easy library (20) and SynBio2Easy (21). SBOL2Easy provides the streamlined functions for generating and manipulating SBOL designs by utilizing the lower-level methods of LibSBOLj library (v2.4.0) (22, 23). The SBOL2Easy library could be easily reused by other projects that would like to utilize some of the implemented operations like, for example, flattening of the designs. The SynBio2Easy application is a Command Line Interface (CLI), the presentation layer that wraps around SBOL2Easy and is responsible for user interaction. It is created with the Spring Boot (24) framework that provides a standardized application base. Apache Maven (25) was used to manage the software build and project dependencies. Docker (26) and the SynBioHub Docker images (27) were used to provide a SynBioHub platform for testing. The tool was tested with version 1.6.0 of the SynBioHub platform API (28). The generated designs were deposited into the public instance of SynBioHub (https://synbiohub.org).

## 3. Results

### 3.1 SynBio2Easy

The SynBio2Easy CLI application enables users to perform batch creation, deposit and update operations on synthetic biology designs encoded as SBOL ComponentDefinitions (29). The tool can either generate and modify SBOL documents and components by reading and writing SBOL files or it can be used to interact directly with a target SynBioHub server to upload new designs or update existing components. ‘Batch’ parameters for particular properties belonging to individual designs are recorded in MS Excel.

The Java programming language was selected for development of the software because it has proven to be very easy to maintain over time, and it is also the primary implementation technology for SBOL 2 libraries and SynBioHub itself. The application was built using the Spring Boot framework, providing numerous advantages including an architecture based on dependency injection that promotes decoupling and simplifies testing. This framework also comes with the benefit that the application can be easily further developed into a web-based graphical UI by extension of the existing code base.

**Figure 1.**
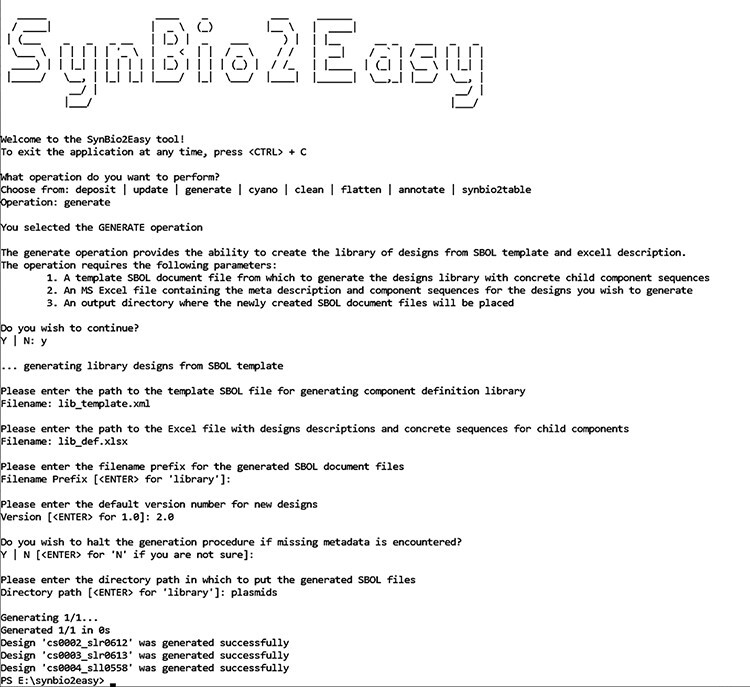
SynBio2Easy Command Line Interface with a guided prompt.

Although the software is a command line tool, it guides the user through each scenario by prompting with a series of questions as seen in the screenshot (Figure 1). The application also supports specification of all parameters as standard command line options, so that it can be easily integrated with scripts or as part of larger workflows. A detailed description of the available commands and options is provided in the README.md file of the SynBio2Easy repository (21). A collection of SBOL documents and Excel metadata input files are also provided in the ‘examples’ folder; the ‘README.md’ in this folder contains an end2end scenario that demonstrates all the SynBio2Easy options using the provided example files as inputs. We also created a document (SynBio2Easy_use_cases.docx) based on use cases that guides the user through a series of typical scenarios for working with a library of designs.

**Figure 2.**
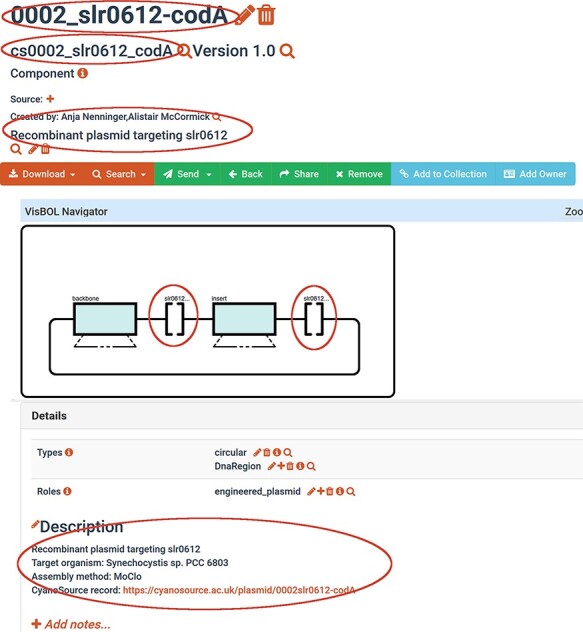
Example of a SynBioHub record (cs0002_slr0612_codA) a CyanoSource plasmid generated from an SBOL template and Excel descriptions. Red circles mark information that was inserted from the Excel input.

**Figure 3.**
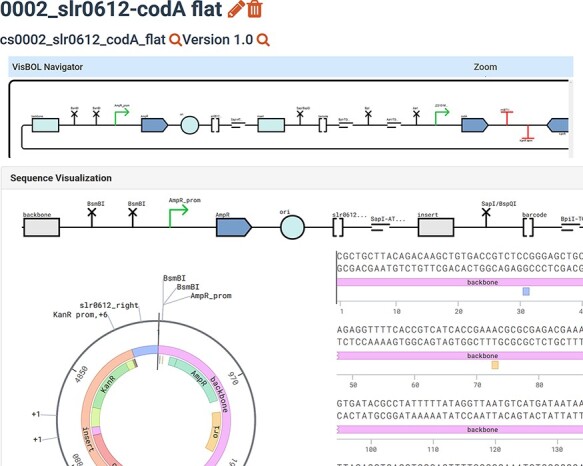
‘Flattened’ version of the same plasmid (cs0002_slr0612_codA) that contains only one fully annotated sequence that permits visualizations. The term ‘flattened’ means that the SBOL subcomponents in a hierarchical design are now mapped as annotations of one linear sequence.

The SynBio2Easy command line tool was used to GENERATE and DEPOSIT (see Table 1) CyanoSource plasmid designs into SynBioHub (Figures 2 and 3). They are available as the ‘cyano_coda_km’ collection accessible via the ‘sharing’ link (30) (shortened URL https://bit.ly/3btmbd4). The use cases were shown to be broadly relevant in a separate project conducted by Trevor Ho *et al.* (31); the ‘Intein_assisted_Bisection_Mapping’ collection generated for this project is publicly available in SynBioHub under the link (32). This collection was created by firstly uploading GenBank files to SynBioHub, then downloading the whole collection as SBOL and enriching it with additional metadata using the CLEAN and ANNOTATE commands of SynBio2Easy (Table 1).

**Table 1.** Available operations of SynBio2Easy

Command	Behavior and example use case
GENERATE	Generates a series of designs based on an SBOL template and ‘concrete’ instance parameters (including sub-components’ sequences) specified in an Excel table Use case: generation of library of similar designs
ANNOTATE	Adds information (e.g. descriptions, authors) to multiple component definitions in an SBOL document using details defined in an Excel table Use case: batch update of designs’ descriptions to change their status to ‘tested’ and add provenance
FLATTEN	Converts a tree of SBOL sub-components in a design into a ‘flattened’ component definition with an annotated linear sequence Use case: create an alternative representation of a plasmid suitable for export to GenBank file format (see Figure 3)
DEPOSIT	Deposits files from a folder(s) into SynBioHub collection(s) Use case: Deposition of a large collection of designs
UPDATE	Adds information (e.g. notes) as well as attachment files to multiple records in SynBioHub using details defined in an Excel table. Unlike ANNOTATE it is an online operation on a server. Use case: attach verified sequences to designs’ descriptions
CLEAN	Removes annotations and namespaces specific to SynBioHub from an SBOL document, so it can be re-uploaded to SynBioHub Use case: quick edit of a SynBioHub collection using a text editor and downloaded XML file
SYNBIO2TABLE	Retrieves identity details of all members of a collection and saves them to an Excel file with headings for metadata columns supported by SynBio2Easy Use case: preparation of input file for the UPDATE operation

For most operations, the tool requires two inputs: ComponentDefinition(s) from an SBOL document or SynBioHub records and metadata directives in MS Excel format that provide details for the actions to be performed on the designs. The Excel table must always contain the ‘display_id’ column, which is used to identify existing ComponentDefinitions (or to create a new one with the specified ID). Other columns named with selected keywords can provide values for designs attributes like name, description text, location of files to attach or definitions of subcomponents that should be concretized from the ‘generic’ template, all depending on the particular operation (see Table 1).

**Figure 4.**
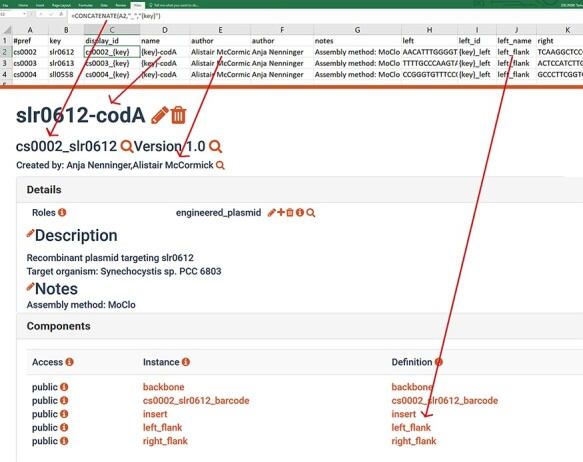
Library definition file (top), for each row a new ComponentDefinition is generated. The metadata are provided in the corresponding columns (e.g. ‘author’), and field values can be dynamically calculated using excel formulas and simple templating. The example of a generated design after it is uploaded to SynBioHub (bottom). Arrows link excel cells to corresponding fields in the SynBioHub record.

Cells in the Excel table can include simple formulas, for example, ‘CONCATENATE($B$2, “is a PCR file”)’ (Figure 4), which will be correctly parsed by the tool. Additionally, the tool supports simple templating, for example, the description text: ‘Attachment {display_id}.xls contains activity data’ would be interpolated by substituting ‘{display_id}’ with the corresponding ComponentDefinition display ID cell’s value in the same row of the table.

Here, we describe a SynBio2Easy ‘GENERATE’ use case: the process of generating a new library of plasmid design records as in stage 1 of the CyanoSource project. In this scenario, the intention is to generate SBOL files describing a library of designs. A template SBOL ComponentDefinition is required, which contains subcomponents without specified sequences, and these subcomponents will vary between members of the library. The concrete sequences for each instance are provided in the MS Excel spreadsheet along with the descriptive metadata (Figure 4, top). The program generates a new SBOL document containing ComponentDefinitions for each Excel record based on the template and the provided details.

The ‘examples’ folder in out GitHub repository (21) contains the example template design in the ‘lib_template.xml’ file, which represents a simple plasmid with ‘left’, ‘right’ and ‘barcode’ subcomponents similar to the real CyanoSource template. Such templates can be generated with a visual tool such as SBOL Designer or SBOL Canvas, although for this project we created the template plasmids programmatically using the SBOL API.

The same folder includes an example of the Excel library definition file ‘lib_def.xlsx’ (Figure 4, top), which provides descriptive metadata like ‘author’, ‘name’, ‘notes’, etc. for each library member. Apart from the metadata, the Excel file contains the concrete sequences for the barcode, left and right subcomponents. Matching between subcomponents and their structures is done using their id and column headers. The additional columns ‘SUBCOMPONENT_id’, ‘SUBCOMPONENT_name’ can be used to customize the label and name of the generated concrete subcomponents. See Figure 4 to observe how the simple variable templating and excel formulas are used to create ‘context’ aware metadata for the individual library members.

Apart from GENERATE, SynBio2Easy supports six other operations: ANNOTATE, FLATTEN, DEPOSIT, UPDATE, CLEAN and SYNBIO2TABLE, which are briefly described in Table 1. We have provided detailed descriptions of all the use cases in the ‘SynBio2Easy_use_cases.docx’ file inside the SynBio2Easy GitHub repository (21). However, we recommend exploring our tool by following our step-by-step quick start instructions from the ‘examples’ folder in GitHub. It describes a simple end-to-end workflow that operates on the provided example files and guides the user through all the available operations of the tool (including ready-to-copy command line instructions).

### 3.2 Limitations

This tool was created to assist us with an actual project, so it is tailored to our particular needs. For example, the batch processing is limited to only instances of ComponentDefinition, flattening works only with sequence locations provided as Range objects, and the tool itself supports only SBOL2.

## 4. Discussion

While developing SynBio2Easy, it became clear that SBOL is a powerful modeling language and that SynBioHub is a highly effective data-sharing platform, both of which are really catering for SBOL experts in the field of synthetic biology. During the initial evaluation of ICE and SynBioHub platforms, one of the authors Anja Nenninger—an experienced biologist—said:

‘I understand ICE more but SynBioHub looks like being the thing of the future.’

However, biologists outside the SBOL community may find a steep learning curve to become acquainted with the standard and the necessary tools. Our SynBio2Easy software reduces barriers to depositing research data into SynBioHub for particular use cases, notably automating similar operations on collections of designs and providing functionality to compensate for the limitations of the current platform. In the following section, we discuss how we overcame some limitations of SynBioHub and give our recommendations for improving adoption of SBOL ecosystem by new users.

### 4.1 Overcoming limitations

Some of the features in SynBio2Easy were implemented in order to compensate for the current usability limitations of the SynBioHub platform.

Visualizations are one of the most important features for end users, which in part motivated our ‘FLATTEN’ command. Feedback from our colleagues has made it clear that biologists ‘love’ the functional glyphs of the designs in SynBioHub. At the same time, they also need to see—and are accustomed to seeing—associated annotated sequences. However, those features are only available for simple designs; once subcomponents are integrated, the visualizations of sequences are no longer accessible. At the start of this project, the subcomponents were rendered in SynBioHub below the top-level design if they also contained a nested subcomponent. Whether it was a bug or a feature we are uncertain, but currently only top-level components are rendered and the complex subcomponents are simply depicted as boxes. This degrades the quality of the records because tedious clicking between designs is required to view the subcomponent sequences. The usefulness of records is therefore greatly limited as the full sequence is not visible.

For the reasons described above, SynBio2Easy offers a ‘FLATTEN’ command, which converts subcomponent tree hierarchies into one annotated sequence (Figures 2 and 3). This feature exists purely to allow the biologist to easily visualize the sequence details, or so they can download the design in GenBank format and edit it in their preferred software such as SnapGene (33). Such flattening cannot be performed for all ‘abstract’ SBOL ComponentDefinitions, but all instantiations that have been physically assembled and whose concrete sequences are known and present can probably be flattened. This process could be easily integrated into SynBioHub as a pre-export step using our Java implementation or before the annotated sequence is rendered by the visualization plugin.

A typical workflow for updating public resources consists of the following actions: downloading, modifying and then uploading again. This workflow is not possible with SynBioHub at present, because the downloaded SBOL files cannot be readily re-uploaded to the server. For this reason, we implemented the ‘CLEAN’ feature of our tool, which automatically removes any annotations specific to SynBioHub in the downloaded SBOL document, enabling re-upload of the file back to the server.

Similarly, the ‘DEPOSIT’ command was implemented to compensate for difficulties with using the SynBioHub web UI to upload large—or sets of multiple—files. Attempting to do so can result in request timeouts and database errors on the back-end server, so the batch upload function provided by SynBio2Easy alleviates these frustrations.

### 4.2 Enhancing SBOL/SynBioHub adoption

The development of this software toolkit proved to be more challenging than anticipated, primarily because we were not directly involved in the SBOL community. Half of our project team have experience in research data management and software development, while the other half are molecular biologists with basic computational and bioinformatics expertise. By reading the SBOL specification, the ‘software’ part of the team learned how to encode, construct and assemble appropriate objects into the correct data structures. However, our limited experience meant that it was not always obvious how to represent the design plasmids. On many occasions, we investigated SBOL and SynBioHub capabilities with a ‘forensic programming’ approach, that is, writing code using the supporting libraries to appraise what the outcome would be, for example, generating component designs with varying attributes and structure and evaluating how they are rendered in SynBioHub.

One helpful element would be a collection of recommended component (29) and module (34) definitions. This collection would showcase how different structures and functions can be expressed and represented in SBOL and how they then look and interlink within SynBioHub. Equipped with these reference templates, new users could use them as the basis of their designs or derive inspiration from them for creating their own entirely new entities. The iGEM collections (35) provide a multitude of examples, but it is not always clear which of these are the most effective and should be considered the best ‘reference’ material upon which to base new designs.

Similarly, there is a shortage of guidance on how to actually encode concrete characteristics of the synthetic constructs; for example, to declare that ‘The target organism is a cyanobacterium’ for a design, the expected function is an ‘XOR gate’ and it is compatible with the Golden Gate Assembly (36, 37) method. Without further guidelines on which namespaces and terms to use from the bio-ontologies, much of the knowledge about designs are likely to become unstructured free text appended in the description field, diminishing the full expressive power of the language.

At the recent Harmony 2021 meeting of SynBioHub developers and users, the idea of ‘minimal information’ for designs was discussed. There is a definite need for recommendations on the structured encoding of standard facts and properties of designs and constituent parts. A list of recommended name spaces and terms are needed, similarly to those currently provided for the ‘role’ and ‘type’ attributes of component definitions.

The flexibility and powerful expressiveness of SBOL should not hinder biologists from using it. For example, to work around the limitation that including subcomponents would prevent users from seeing the entire sequence in the forms of functional glyphs, we could simply generate only ‘flat’ designs equivalent to annotated DNA sequences. Such representations are essentially GenBank files and defeat the purpose of SBOL. Naturally, the UI should not detract from the rich features offered by the SBOL language. Therefore, it would be a very welcome function in the UI if the user could zoom into subcomponent structures from the top-level design and view the functional glyphs belonging to its subcomponents.

We found the SynBioHub API easy to use and basic tasks are well documented, with practical examples for Shell scripting, Python and JavaScript languages. However, it would be beneficial to adopt a fully RESTful (38) API design perhaps based on a standard specification such as the OpenAPI (39), especially to reduce the number of requests for the update operation.

On multiple occasions, the public SynBioHub instances manifested glitches preventing it from being used (for instance, rendering loops, failing search engine and timeouts). It is important that any stability issues with the platform are detected early and remedied since users who have a poor experience might be discouraged from returning to the platform in the future.

## 5. Conclusions

At the recent HARMONY 2021, Professor Chris J. Myers and his team from the University of Colorado Boulder presented their Excel2SBOL (18) tool, which performs functionality similar to that of our tool. This indicates that the previously described shortcomings have also been identified by other researchers and their use cases. While the software that we produced does address the missing functionalities and limitations in the current SBOL/SynBioHub ecosystem, we hope SynBio2Easy will become redundant once the new versions of SBOL 3.0 and SynBioHub 2.0 are delivered.

Ultimately, for successful adoption of SBOL, SynBioHub should handle all aspects of working with the designs, from specifying their structure, annotating sequences to online editing of all the information. The whole synthetic biology community should support SynBioHub, or an equivalent, that can provide a combination of CAD-like features with a powerful search engine, to become the default tool for synthetic biologists.

In the meantime, we hope that SynBio2Easy and similar tools will help in widening adoption of the SBOL standard and incorporation of SynBioHub into daily research workflows, even by non-expert users.

## Data Availability

The version 1.7.0 of SynBio2Easy tool, which is described in this manuscript, has been deposited to Zenodo as https://doi.org/10.5281/zenodo.5719362. The source code for the command line tool SynBio2Easy is available in GitHub: https://github.com/BioRDM/synbio2easy. The actual implementation of batch operations on SBOL documents is decoupled from the command line driver and available as the sbol2easy project: https://github.com/BioRDM/sbol2easy.
